# Trends in Opioid Toxicity–Related Deaths in the US Before and After the Start of the COVID-19 Pandemic, 2011-2021

**DOI:** 10.1001/jamanetworkopen.2023.22303

**Published:** 2023-07-07

**Authors:** Tara Gomes, Shaleesa Ledlie, Mina Tadrous, Muhammad Mamdani, J. Michael Paterson, David N. Juurlink

**Affiliations:** 1 Li Ka Shing Knowledge Institute, St. Michael’s Hospital, Toronto, Ontario, Canada; 2 Leslie Dan Faculty of Pharmacy, University of Toronto, Toronto, Ontario, Canada; 3 Institute for Health Policy, Management and Evaluation, University of Toronto, Toronto, Ontario, Canada; 4 ICES, Toronto, Ontario, Canada; 5 Sunnybrook Research Institute, Toronto, Ontario, Canada

## Abstract

**Importance:**

Opioid-related harms constitute a major public health crisis in the US, and this crisis has worsened during the COVID-19 pandemic.

**Objectives:**

To characterize the societal burden of unintentional opioid-related deaths in the US and describe changing mortality patterns during the COVID-19 pandemic.

**Design, Setting, and Participants:**

A serial cross-sectional study examined all unintentional opioid-related deaths in the US, evaluated annually from calendar years 2011 to 2021.

**Main Outcomes and Measures:**

The public health burden of opioid toxicity–related deaths was estimated in 2 ways. First, the proportion of all deaths that were attributable to unintentional opioid toxicity by year (2011, 2013, 2015, 2017, 2019, and 2021) and age group (15-19, 20-29, 30-39, 40-49, 50-59, and 60-74 years) were calculated, using age-specific estimates of all-cause mortality as the denominator. Second, the total years of life lost (YLL) due to unintentional opioid toxicity was estimated, overall and by sex and age group, for each year studied.

**Results:**

Among the 422 605 unintentional deaths due to opioid toxicity between 2011 and 2021, the median age of the individuals was 39 (IQR, 30-51) years, and 69.7% were male. The number of unintentional deaths due to opioid toxicity increased 289% over the study period, from 19 395 (2011) to 75 477 (2021). Similarly, the percentage of all deaths that were attributed to opioid toxicity increased from 1.8% in 2011 to 4.5% in 2021. By 2021, opioid toxicity was responsible for 10.2% of all deaths among those aged 15 to 19 years, 21.7% of deaths among those aged 20 to 29 years, and 21.0% of deaths among those aged 30 to 39 years. The YLL due to opioid toxicity increased 276% over the study period, from 777 597 in 2011 to 2 922 497 in 2021. While YLL plateaued between 2017 (7.0 YLL per 1000) and 2019 (7.2 YLL per 1000), it increased by 62.9% between 2019 and 2021 coincident with the COVID-19 pandemic, reaching 11.7 YLL per 1000 population. This relative increase was similar across all age groups and sexes with the exception of those aged 15 to 19 years, in whom the YLL nearly tripled, from 1.5 to 3.9 YLL per 1000 population.

**Conclusions and Relevance:**

In this cross-sectional study, deaths due to opioid toxicity increased substantially during the COVID-19 pandemic. By 2021, 1 of every 22 deaths in the US was attributable to unintentional opioid toxicity, underscoring the urgent need to support people at risk of substance-related harm, particularly men, younger adults, and adolescents.

## Key Points

**Question:**

What is the societal burden of unintentional opioid-related deaths in the US, and has it changed during the COVID-19 pandemic?

**Findings:**

In this cross-sectional study of 422 605 unintentional deaths due to opioid toxicity, the years of life lost increased more than 3-fold, from 777 597 to 2 922 497, between 2011 and 2021. By 2021, 1 of every 22 deaths in the US was attributable to unintentional opioid toxicity.

**Meaning:**

The findings of this study suggest that public health impacts of opioid-related toxicity in the US are broad and have worsened during the pandemic, underscoring the urgent need to support people at risk of substance-related harm, particularly men, younger adults, and adolescents.

## Teaser

This cross-sectional study examines changes in death rates due to unintentional opioid toxicity between 2011 and 2021.

## Introduction

Over the past 2 decades, opioid-related harms have emerged as a leading public health crisis in North America. In the US, the number of deaths due to opioid toxicity has increased from 21 088 in 2010 to nearly 70 000 in 2020, with the increase due primarily to fentanyl and its analogues.^1,2^ Younger adults represent a disproportionate number of these deaths,^3^ with early loss of life imparting a major toll on public health.^1,3^ Despite early signals that rates of death due to opioid toxicity slowed between 2017 and 2019,^1^ the COVID-19 pandemic carried with it major changes in societal priorities and access to health services that, along with the increasing dangers of the unregulated drug supply, were anticipated to aggravate opioid-related harms.^4,5^ In 2020, the US Centers for Disease Control and Prevention (CDC) reported 68 630 opioid-related deaths, a 47% increase from 2018.^1^

In 2016, deaths due to opioid toxicity resulted in an estimated 1.68 million years of life lost (YLL) in the US,^3^ and in 2019, it was estimated that more than half of all deaths due to drug toxicity worldwide occurred in the US.^6^ We sought to characterize the growing societal burden of unintentional opioid-related deaths in the US and examine patterns during the COVID-19 pandemic and across demographic subgroups of the population.

## Methods

### Study Design and Population

We conducted a serial cross-sectional study by extracting annual counts of deaths due to unintentional opioid toxicity in the US between calendar years 2011 and 2021. Informed consent was not required given the deidentified, aggregated nature of the data. The study received an exemption from the Unity Health Toronto Research Ethics Board, Toronto, Ontario, Canada. This study follows the Strengthening the Reporting of Observational Studies in Epidemiology (STROBE) reporting guideline for cross-sectional studies.

### Data Sources and Outcome Definition

We used data from the CDC Wide-Ranging Online Data for Epidemiologic Research (WONDER) Multiple Cause of Death database, which captures national-level mortality and population data for all US residents using information recorded on death certificates (eTable 1 in Supplement 1).^7^ Our main outcome was unintentional deaths in which prescribed or unregulated (principally heroin and analogues of fentanyl) opioids contributed to death. Consistent with CDC standards,^1^ we defined opioid toxicity–related deaths as those in which an underlying cause of death due to unintentional drug poisoning and a multiple cause of death code related to an opioid were recorded (eTable 2 in Supplement 1). To avoid data gaps due to suppressed data, we excluded all deaths among people younger than 15 years and older than 74 years.

### Burden of Unintentional Opioid Toxicity–Related Deaths

We defined the burden of unintentional deaths due to opioid toxicity in 2 ways. First, we determined the proportion of all deaths attributable to unintentional opioid toxicity by year and age group (15-19, 20-29, 30-39, 40-49, 50-59, and 60-74 years) using age-specific estimates of all-cause mortality as the denominator. Second, we used methods adapted from the Global Burden of Disease Study^8^ to estimate the YLL due to unintentional opioid toxicity (eTable 3 in Supplement 1). We aggregated YLL by sex and age group for each year of the study period, reported as absolute numbers and population-adjusted rates.

In a sensitivity analysis to explore and contrast the findings with deaths related to COVID-19, we replicated the overall estimates of the proportions of deaths attributable to unintentional opioid toxicity, excluding all COVID-19–related deaths (underlying cause of death *International Statistical Classification of Diseases and Related Health Problems, 10th Revision* code U07.1) from the denominator in 2020 and 2021. Additionally, in a secondary analysis, we calculated the YLL due to COVID-19 in 2020 and 2021, using methods consistent with those outlined above.

### Statistical Analysis

We used descriptive statistics to summarize demographic characteristics of unintentional deaths due to opioid toxicity and the Cochrane-Armitage test for trend to compare the percentage of deaths attributable to opioids by age group over time. All analyses used SAS, version 9.4 (SAS Institute) or Microsoft Excel (Microsoft Corp), and a 2-sided, type I error rate of .05 was the threshold for statistical significance.

## Results

**Figure 1.**
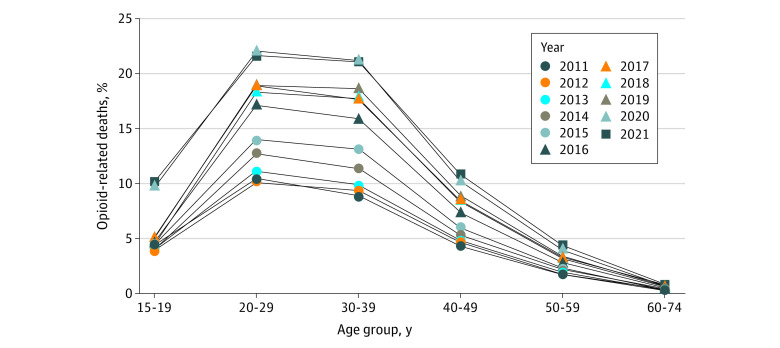
Percentage of All-Cause Deaths Attributable to Unintentional Opioid Toxicity The percentage of all-cause deaths attributable to unintentional opioid toxicity increased between 2011 and 2021 across all age groups, with large increases observed between 2019 and 2020, coincident with the COVID-19 pandemic.

During the study period, among the 422 605 unintentional deaths due to opioid toxicity, the median age of the individuals was 39 (IQR, 30-51) years, 69.7% were male, and 30.3% were female. The number of unintentional deaths due to opioid toxicity increased 289% over the study period, from 19 395 (83.8 per million) in 2011 to 75 477 (302.9 per million) in 2021. The percentage of all deaths attributable to opioid toxicity increased from 1.8% in 2011 to 4.5% in 2021 (Figure 1), with a statistically significant trend observed in all age groups. For example, among those aged 30 to 39 years, the proportion increased from 8.9% (2011) to 21.0% (2021) (*P* < .001). By 2021, more than 20% of all deaths among individuals aged 20 to 29 years (21.7%) and 30 to 39 years (21.0%) were due to opioid toxicity; however, the largest relative increase occurred among those aged 15 to 19 years, in whom the proportion of deaths attributable to opioid toxicity more than doubled, from 5.0% in 2019 to 10.2% in 2021. In the sensitivity analysis excluding COVID-19–related deaths from the denominator, the percentage of deaths attributable to opioid toxicity increased to 5.3% in 2021.

**Figure 2.**
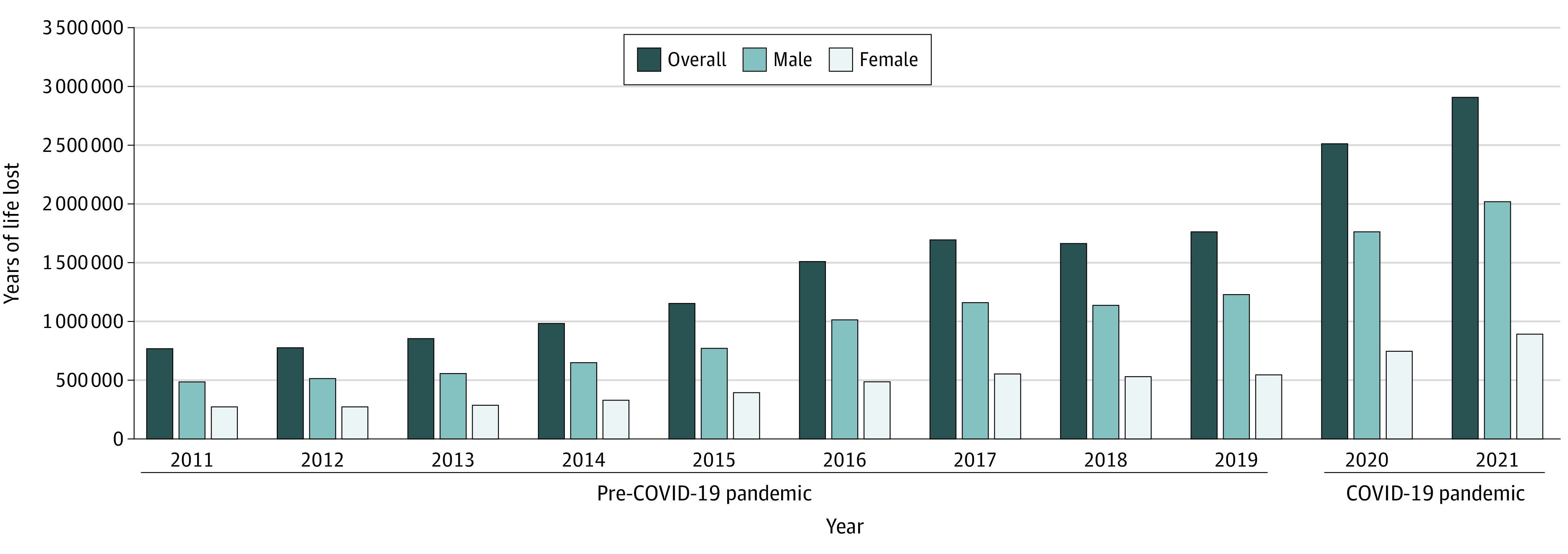
Years of Life Lost to Unintentional Opioid Toxicity in the US Years of life lost were calculated using methods adapted from the Global Burden of Disease Study.^8^ The years of life lost remained higher in males throughout the study.

The YLL due to opioid toxicity in the US increased substantially over the study period, from 777 597 YLL (2011) to 2 922 497 YLL (2021)—a relative increase of 276% (Table 1; eTable 4 in Supplement 1). However, this increase was not consistent over the study period. While YLL increased from 2011 to 2017, it plateaued between 2017 (7.0 YLL per 1000 population) and 2019 (7.2 YLL per 1000 population) (Figure 2, Table 1). Over the ensuing 2 years, and coincident with the COVID-19 pandemic, the population-adjusted rate of YLL increased by 62.9%, reaching 11.7 YLL per 1000 population in 2021. This pattern was similar across age groups and sexes except among those aged 15 to 19 years, in whom the YLL nearly tripled, from 1.5 to 3.9 YLL per 1000 population (from 31 097 to 83 193 YLL) (Table 1).

**Table 1.** Years of Life Lost Due to Unintentional Opioid Toxicity in the US by Age and Sex, 2019 to 2021

Age groups, y	2019, No.				2020, No.				2021, No.			
	Unintentional opioid-related deaths	Deaths per million	YLL	YLL per 1000	Unintentional opioid-related deaths	Deaths per million	YLL	YLL per 1000	Unintentional opioid-related deaths	Deaths per million	YLL	YLL per 1000
Overall	45 315	184.9	1 772 030	7.2	63 946	259.9	2 521 813	10.2	75 477	302.9	2 922 497	11.7
15-19	515	24.5	31 097	1.5	1196	57.1	72 260	3.4	1371	63.6	83 193	3.9
20-29	8786	194.6	468 243	10.4	12 377	276.1	660 835	14.7	13 035	296.8	695 949	15.8
30-39	13 234	299.6	596 845	13.5	18 831	421.6	848 304	19.0	21 741	478.9	979 090	21.6
40-49	9623	238.7	347 225	8.6	13 741	341.1	497 694	12.4	16 650	407.2	604 488	14.8
50-59	8811	208.0	242 351	5.7	11 768	280.2	322 987	7.7	14 581	343.3	399 710	9.4
60-74	4346	83.5	86 269	1.7	6033	113.1	119 732	2.2	8099	147.5	160 068	2.9
Males	32 041	264.1	1 224 382	10.1	45 852	376.4	1 765 682	14.5	53 797	433.7	2 030 032	16.4
15-19	370	34.4	21 846	2.0	879	82.2	51 957	4.9	962	87.3	56 926	5.2
20-29	6388	276.9	332 428	14.4	9120	398.1	475 773	20.8	9475	423.9	493 409	22.1
30-39	9442	424.6	414 342	18.6	13 628	605.5	597 869	26.6	15 685	683.8	688 162	30.0
40-49	6878	344.0	241 137	12.1	9756	488.3	342 525	17.1	11 853	579.1	417 216	20.4
50-59	5947	286.9	157 236	7.6	8167	397.2	215 820	10.5	10 037	476.4	264 712	12.6
60-74	3016	122.8	57 394	2.3	4302	171.0	81 738	3.2	5785	220.8	109 606	4.2
Females	13 274	107.3	547 648	4.4	18 094	145.6	756 130	6.1	21 680	173.3	892 466	7.1
15-19	145	14.1	9251	0.9	317	30.9	20 303	2.0	409	38.8	26 266	2.5
20-29	2398	108.6	135 815	6.2	3257	148.6	185 062	8.4	3560	165.1	202 540	9.4
30-39	3792	172.9	182 503	8.3	5203	234.8	250 435	11.3	6056	269.6	290 928	13.0
40-49	2745	135.0	106 088	5.2	3985	196.3	155 169	7.6	4797	235.0	187 272	9.2
50-59	2864	132.4	85 114	3.9	3601	168.0	107 167	5.0	4544	212.3	134 998	6.3
60-74	1330	48.4	28 876	1.1	1731	61.4	37 994	1.3	2314	80.6	50 463	1.8

Abbreviation: YLL, years of life lost.

In 2021, the total burden of deaths due to opioid toxicity was highest among men (16.4 vs 7.1 YLL per 1000 for men vs women) and those aged 30 to 39 years (21.6 per 1000). The highest burden was evident among men aged 30 to 39 years, with 15 685 individuals dying from opioid toxicity in 2021, leading to 688 162 YLL (30.0 YLL per 1000 population).

In the secondary analysis, there were 3 066 440 YLL due to COVID-19 in 2020, increasing to 5 512 380 YLL in 2021 (Table 2). Years of life lost was generally higher among men compared with women and among those aged 40 years and older. Among individuals aged 15 to 39 years, the YLL due to opioid toxicity in both 2020 and 2021 were higher than those attributable to COVID-19.

**Table 2.** Years of Life Lost to COVID-19 in the US by Age and Sex, 2020 and 2021

Age groups, y	2020, No.				2021, No.			
	COVID-19 related deaths	Deaths per million	YLL	YLL per 1000	COVID-19 related deaths	Deaths per million	YLL	YLL per 1000
Overall	144 165	585.8	3 066 440	12.5	236 862	950.7	5 512 380	22.1
15-19	118	5.6	7192	0.3	351	16.3	21 603	1.0
20-29	1175	26.2	62 892	1.4	3149	71.7	168 927	3.8
30-39	3729	83.5	166 078	3.7	10 330	227.5	462 340	10.2
40-49	10 410	258.5	367 648	9.1	24 412	597.1	870 494	21.3
50-59	26 997	642.8	723 631	17.2	53 384	1256.9	1 445 540	34.0
60-74	101 736	1907.0	1 738 999	32.6	145 236	2645.4	2 543 476	46.3
Males	91 239	749.0	1 871 371	15.4	142 827	1151.4	3 198 167	25.8
15-19	59	5.5	3449	0.3	215	19.5	12 828	1.2
20-29	763	33.3	39 550	1.7	1964	87.9	101 732	4.6
30-39	2500	111.1	107 682	4.8	6491	283.0	280 329	12.2
40-49	6953	348.0	236 695	11.8	15 237	744.4	521 104	25.5
50-59	17 751	863.2	456 289	22.2	33 525	1591.4	867 473	41.2
60-74	63 213	2512.3	1 027 705	40.8	85 395	3259.1	1 414 701	54.0
Females	52 926	425.9	1 195 069	9.6	94 035	751.7	2 314 213	18.5
15-19	59	5.7	3743	0.4	136	12.9	8775	0.8
20-29	412	18.8	23 342	1.1	1185	55.0	67 194	3.1
30-39	1229	55.5	58 396	2.6	3839	170.9	182 011	8.1
40-49	3457	170.3	130 953	6.5	9175	449.4	349 390	17.1
50-59	9246	431.4	267 342	12.5	19 859	927.6	578 068	27.0
60-74	38 523	1366.6	711 294	25.2	59 841	2085.1	1 128 776	39.3

Abbreviation: YLL, years of life lost.

## Discussion

In 2021, 1 in 22 deaths in the US was attributable to unintentional opioid toxicity, resulting in nearly 3 million YLL. This burden was most pronounced among men aged 30 to 39 years but has been increasing, especially rapidly among those aged 15 to 19 years, with 1 in 10 deaths now opioid related. The overall burden increased considerably during the COVID-19 pandemic, increasing 63% between 2019 and 2021 despite stabilizing from 2017 to 2019.^1^

These findings highlight the enormous societal burden imparted by the overdose crisis in the US over the past decade, particularly among younger adults, a demographic cohort disproportionately impacted by substance-related harms.^1,3,9^ In addition, despite historically lower rates of opioid-related deaths among adolescents, this demographic cohort witnessed a more than doubling in death rates and YLL during the COVID-19 pandemic. Furthermore, the absolute number of unintentional deaths due to opioid toxicity and the associated YLL among those younger than 40 years far exceeded those attributable to COVID-19 in both 2020 and 2021, indicating the distinct impact of drug toxicity–related deaths among younger individuals. This aligns with previous observations of accelerating substance-related harm among adolescents^10^ and warrants further attention and expansion of harm reduction and treatment programs tailored to this demographic population.

It is important to place the findings of this study in context against other causes of death in the US. Specifically, these findings suggest that the YLL from unintentional opioid toxicity in 2020 (2 521 813 YLL) was comparable to the YLL from COVID-19 that same year (3 066 440 YLL) and amounted to 53% of the YLL attributable to COVID-19 in 2021 (2 922 497 YLL/5 512 380 YLL). Similarly, compared with estimates for other causes of death from the 2019 Global Burden of Disease study, the YLL attributable to opioid toxicity among those aged 15 to 74 years in the US (1 772 030 YLL in 2019) far exceeded those attributable to diabetes (1 115 879 YLL), road injuries (1 602 101 YLL), and stroke (1 442 702 YLL) in this population.^11^

### Limitations

This study has limitations. Death investigations in the US are managed at the state level and may involve a medical examiner system, coroner system, or both.^12^ Therefore, while our definitions align with those of the CDC, some ascertainment bias may be present. Furthermore, cause of death data are not yet available for 2022; future research should explore the ongoing influence of the COVID-19 pandemic on drug toxicity–related deaths in the US.

## Conclusions

The crisis of deaths due to opioid toxicity across the US worsened substantially during the COVID-19 pandemic, with 1 in 22 deaths in 2021 attributable to unintentional opioid toxicity. The findings noted in this cross-sectional study underscore the urgent need to support people at risk of substance-related harm, particularly men, younger adults, and adolescents.

## Notes

Supplement 1. **eTable 1.** Databases Used to Capture All-Cause and Opioid-Toxicity Deaths in the United States **eTable 2.** *International Classification of Diseases and Related Health Problems, 10th Revision* Diagnosis Codes and Descriptions **eTable 3.** Details on Calculation of Years of Life Lost **eTable 4.** Years of Life Lost Due to Accidental Opioid Toxicities in the United States. Overall and by Age and Sex, 2011 to 2018

Supplement 2.
Data Sharing Statement

## References

[1] National Institute of Drug Abuse. Drug overdose death rates. 2022. Accessed February 2, 2023. https://nida.nih.gov/research-topics/trends-statistics/overdose-death-rates#:~:text=Opioid%2Dinvolved%20overdose%20deaths%20rose

[2] Spencer MR, Minino AM, Warner M. Drug overdose deaths in the United States, 2001–2021. 2022. NCHS Data Brief. 2022;(457):1-8. 36598401

[3] Gomes T, Tadrous M, Mamdani MM, Paterson JM, Juurlink DN. The burden of opioid-related mortality in the United States. JAMA Netw Open. 2018;1(2):e180217. doi:10.1001/jamanetworkopen.2018.0217 30646062 PMC6324425

[4] Canadian Centre on Substance Use and Addiction. Changes related to COVID-19 in the illegal drug supply and access to services, and resulting health harms. 2020. Accessed February 2, 2023. https://www.ccsa.ca/changes-related-covid-19-illegal-drug-supply-and-access-services-and-resulting-health-harms

[5] Chacon NC, Walia N, Allen A, . Substance use during COVID-19 pandemic: impact on the underserved communities. Discoveries (Craiova). 2021;9(4):e141. doi:10.15190/d.2021.20 35261922 PMC8896880

[6] Diseases GBD, Injuries C; GBD 2019 Diseases and Injuries Collaborators. Global burden of 369 diseases and injuries in 204 countries and territories, 1990-2019: a systematic analysis for the Global Burden of Disease Study 2019. Lancet. 2020;396(10258):1204-1222. doi:10.1016/S0140-6736(20)30925-9 33069326 PMC7567026

[7] Centers for Disease Control and Prevention. National Center for Health Statistics mortality data on CDC Wonder. 2022. Accessed February 2, 2023. https://wonder.cdc.gov/mcd.html

[8] Department of Information Evidence and Research. *Who Methods and Data Sources for Global Burden of Disease Estimates 2000-2015*. World Health Organization; 2017.

[9] Gomes T, Kitchen SA, Murray R. Measuring the burden of opioid-related mortality in Ontario, Canada, during the COVID-19 pandemic. JAMA Netw Open. 2021;4(5):e2112865. doi:10.1001/jamanetworkopen.2021.12865 34037734 PMC8155822

[10] Kim S, Rajack N, Mondoux SE, Tardelli VS, Kolla NJ, Le Foll B. The COVID-19 impact and characterization on substance use–related emergency department visits for adolescents and young adults in Canada: practical implications. J Eval Clin Pract. 2023;29(3):447-458. doi:10.1111/jep.13817 36752167

[11] Institute for Health Metrics and Evaluation. Global Burden of Disease Collaborative Network. Global Burden of Disease Study. 2020. Accessed February 2, 2023. https://vizhub.healthdata.org/gbd-results/

[12] Centers for Disease Control and Prevention. Death Investigation System. 2019. Accessed February 2, 2023. https://www.cdc.gov/phlp/publications/coroner/death.html

