# Supplementary material for: Trends in Opioid Toxicity–Related Deaths in the US Before and After the Start of the COVID-19 Pandemic, 2011-2021
Source: JAMA Netw Open. 2023 Jul 7;6(7):e2322303. doi: 10.1001/jamanetworkopen.2023.22303 (PMC10329206; doi:10.1001/jamanetworkopen.2023.22303)
Supplement: Supplement 1. — eTable 1. Databases Used to Capture All-Cause and Opioid-Toxicity Deaths in the United States eTable 2. International Classification of Diseases and Related Health Problems, 10th Revision Diagnosis Codes and Descriptions eTable 3. Details on Calculation of Years of Life Lost eTable 4. Years of Life Lost Due to Accidental Opioid Toxicities in the United States. Overall and by Age and Sex, 2011 to 2018 [file jamanetwopen-e2322303-s001.pdf]

Supplementary Online Content

Gomes T, Ledlie S, Tadrous M, Mamdani M, Paterson JM, Juurlink DN. Trends in opioid toxicity–related deaths in the US before and after the start of the COVID-19 pandemic, 2011-2021. *JAMA Netw Open*. 2023;6(7):e2322303. doi:10.1001/jamanetworkopen.2023.22303

eTable 1. Databases Used to Capture All-Cause and Opioid-Toxicity Deaths in the United States

eTable 2. *International Classification of Diseases and Related Health Problems, 10th Revision* Diagnosis Codes and Descriptions

eTable 3. Details on Calculation of Years of Life Lost

eTable 4. Years of Life Lost Due to Accidental Opioid Toxicities in the United States. Overall and by Age and Sex, 2011 to 2018

This supplementary material has been provided by the authors to give readers additional information about their work.

© 2023 Gomes T et al. *JAMA Network Open.*

eTable 1: Databases used to capture all-cause and opioid-toxicity deaths in the United States.

Data Element	Data Source	Description
Opioid Toxicity Deaths	U.S. CDC Wonder Database Multiple Cause of Death Online Database	This database captures national-level mortality and population data for all U.S. residents using information recorded on death certificates including a single underlying cause of death and up to 20 additional multiple causes of death. Data are reported by single-year and 5-year age groups, sex, and calendar year, and are suppressed in cases where fewer than 10 deaths occur within a stratum.
U.S. Population Estimates	U.S. CDC Wonder Database Multiple Cause of Death Online Database	This database captures population estimates for the U.S. based on the U.S. Census Bureau estimates of national resident populations as of July 1st of each year.

© 2023 Gomes T et al. *JAMA Network Open.*

eTable 2: ***International Classification of Diseases and Related Health Problems, 10th Revision*** **Diagnosis Codes and Descriptions**

ICD-10 Code	Descriptions
X40	Accidental poisoning by and exposure to nonopioid analgesics, antipyretics and antirheumatics
X41	Accidental poisoning by and exposure to antiepileptic, sedative-hypnotic, antiparkinsonism and psychotropic drugs, not elsewhere classified
X42	Accidental poisoning by and exposure to narcotics and psychodysleptics [hallucinogens], not elsewhere classified
X43	Accidental poisoning by and exposure to other drugs acting on the autonomic nervous system
X44	Accidental poisoning by and exposure to other and unspecified drugs, medicaments and biological substances
T40.0	Opium
T40.1	Heroin
T40.2	Other Opioids
T40.3	Methadone
T40.4	Other Synthetic Narcotics
T40.6	Other and Unspecified Narcotics

© 2023 Gomes T et al. *JAMA Network Open.*

eTable 3: Details on calculation of Years of Life Lost

YLL Component	Definition
**5-year life expectancy tables**	We calculated the YLL using 2019 standard 5-year life expectancy tables published by the CDC*
**Discounting and Age Weights**	We did not apply discounting or age weights to YLL estimates to align with World Health Organizations guidance**
**Average age at death**	We determined the average age at death for each 5-year age group using the single-year age-stratified estimates of accidental opioid-toxicity deaths accessed from the U.S. CDC Wonder Database Multiple Cause of Death Online Database

**Centers for Disease Control and Prevention. National Center for Health Statistics Multiple cause of death files, 1999-2021. CDC Wonder Online Database. https://wonder.cdc.gov/mcd.html. Published 2022. Accessed February 2, 2023*

***Department of Information Evidence and Research. WHO Methods and Data Sources for Global Burden of Disease Estimates 2000-2015. Geneva, Switzerland. 2017.*

© 2023 Gomes T et al. *JAMA Network Open.*

eTable 4: Years of Life Lost due to Accidental Opioid Toxicities in the United States. Overall and by Age and Sex, 2011 to 2018

	2011				2012			
**Age Groups**	**Accidental Opioid-Related Deaths**	**Deaths Per Million**	**YLLs**	**YLL per 1000**	**Accidental Opioid-Related Deaths**	**Deaths Per Million**	**YLLs**	**YLL per 1000**
**Overall**	19,395	83.8	777,597	3.4	19,782	84.7	785,921	3.4
**15-19**	492	22.7	29,641	1.4	379	17.7	22,778	1.1
**20-29**	4,106	94.5	220,718	5.1	4,064	92.4	218,409	5.0
**30-39**	4,526	112.9	205,212	5.1	4,779	118.3	216,721	5.4
**40-49**	4,992	115.6	180,580	4.2	4,994	116.9	180,333	4.2
**50-59**	4,283	100.0	120,978	2.8	4,389	101.2	123,700	2.9
**60-74**	996	24.7	20,468	0.5	1177	28.2	23,980	0.6
Males	12,681	110.7	505,423	4.4	12,969	112.2	510,897	4.4
**15-19**	377	33.9	22,281	2.0	296	27.0	17,496	1.6
**20-29**	3,094	140.2	162,788	7.4	3,010	134.4	158,338	7.1
**30-39**	3,105	154.9	136,862	6.8	3,306	163.6	145,667	7.2
**40-49**	3,002	140.2	104,188	4.9	3,065	144.7	106,498	5.0
**50-59**	2,545	121.9	68,337	3.3	2,596	122.8	69,408	3.3
**60-74**	558	29.3	10,967	0.6	696	35.3	13,489	0.7
Females	6,714	57.4	272,174	2.3	6,813	57.8	275,024	2.3
**15-19**	115	10.9	7,361	0.7	83	8.0	5,282	0.5
**20-29**	1012	47.4	57,930	2.7	1054	48.8	60,071	2.8
**30-39**	1,421	70.8	68,351	3.4	1,473	73.0	71,053	3.5
**40-49**	1,990	91.4	76,392	3.5	1,929	89.6	73,835	3.4
**50-59**	1,738	79.2	52,641	2.4	1,793	80.8	54,292	2.4
**60-74**	438	20.6	9,500	0.4	481	21.8	10,491	0.5

© 2023 Gomes T et al. *JAMA Network Open.*

	2013				2014			
**Age Groups**	**Accidental Opioid-Related Deaths**	**Deaths Per Million**	**YLLs**	**YLL per 1000**	**Accidental Opioid-Related Deaths**	**Deaths Per Million**	**YLLs**	**YLL per 1000**
**Overall**	21,626	91.8	852,807	3.6	24,921	104.7	983,772	4.1
**15-19**	369	17.4	22,206	1.0	365	17.3	21,995	1.0
**20-29**	4,471	100.8	239,896	5.4	5,246	116.8	281,119	6.3
**30-39**	5,155	126.1	233,821	5.7	6,241	150.6	282,405	6.8
**40-49**	5,160	122.7	186,458	4.4	5,651	136.2	204,435	4.9
**50-59**	4,978	113.8	139,808	3.2	5,617	127.4	157,127	3.6
**60-74**	1493	34.4	30,618	0.7	1801	40.1	36,691	0.8
Males	14,252	122.2	558,550	4.8	16,481	140.0	645,725	5.5
**15-19**	279	25.7	16,464	1.5	261	24.2	15,341	1.4
**20-29**	3,371	148.9	176,972	7.8	3,899	170.3	204,313	8.9
**30-39**	3,599	175.8	158,955	7.8	4,407	212.4	194,429	9.4
**40-49**	3,192	153.0	110,780	5.3	3,506	170.5	121,990	5.9
**50-59**	2,927	137.1	78,121	3.7	3,373	156.7	89,616	4.2
**60-74**	884	43.2	17,259	0.8	1035	48.8	20,037	0.9
Females	7,374	62.0	294,258	2.5	8,440	70.2	338,047	2.8
**15-19**	90	8.7	5,742	0.6	104	10.1	6,654	0.6
**20-29**	1100	50.6	62,924	2.9	1347	61.2	76,806	3.5
**30-39**	1,556	76.3	74,867	3.7	1,834	88.6	87,976	4.2
**40-49**	1,968	92.8	75,679	3.6	2,145	102.6	82,446	3.9
**50-59**	2,051	91.6	61,687	2.8	2,244	99.5	67,511	3.0
**60-74**	609	26.6	13,360	0.6	766	32.3	16,654	0.7

© 2023 Gomes T et al. *JAMA Network Open.*

	2015				2016			
**Age Groups**	**Accidental Opioid-Related Deaths**	**Deaths Per Million**	**YLLs**	**YLL per 1000**	**Accidental Opioid-Related Deaths**	**Deaths Per Million**	**YLLs**	**YLL per 1000**
**Overall**	29,248	121.8	1,162,133	4.8	37,679	156.0	1,507,795	6.2
**15-19**	450	21.3	27,133	1.3	532	25.2	32,075	1.5
**20-29**	6,223	137.7	332,992	7.4	8,403	185.6	449,296	9.9
**30-39**	7,828	186.2	353,976	8.4	10,490	246.5	474,073	11.1
**40-49**	6,393	155.7	231,177	5.6	7,987	196.5	288,385	7.1
**50-59**	6,173	139.8	172,640	3.9	7,506	171.3	208,121	4.7
**60-74**	2,181	46.8	44,215	0.9	2761	57.4	55,846	1.2
Males	19,638	165.2	772,361	6.5	25,995	217.5	1,026,952	8.6
**15-19**	314	29.1	18,473	1.7	381	35.3	22,458	2.1
**20-29**	4,629	200.6	242,024	10.5	6,249	270.3	326,688	14.1
**30-39**	5,584	265.1	245,953	11.7	7,564	354.4	333,253	15.6
**40-49**	4,114	202	143,214	7	5,320	264.0	185,721	9.2
**50-59**	3,606	167.2	95,727	4.4	4,717	220.3	124,853	5.8
**60-74**	1,391	63.2	26,971	1.2	1764	77.7	33,978	1.5
Females	9,610	79.2	389,772	3.2	11,684	95.8	480,843	3.9
**15-19**	136	13.2	8,660	0.8	151	14.6	9,616	0.9
**20-29**	1,594	72	90,968	4.1	2154	97.2	122,608	5.5
**30-39**	2,244	106.9	108,023	5.1	2,926	137.9	140,819	6.6
**40-49**	2,279	110.1	87,963	4.2	2,667	130.2	102,664	5.0
**50-59**	2,567	113.7	76,914	3.4	2,789	124.5	83,268	3.7
**60-74**	790	32.1	17,244	0.7	997	39.2	21,868	0.9

© 2023 Gomes T et al. *JAMA Network Open.*

Age Groups	2017				2018			
	**Accidental Opioid-Related Deaths**	**Deaths Per Million**	**YLLs**	**YLL per 1000**	**Accidental Opioid-Related Deaths**	**Deaths Per Million**	**YLLs**	**YLL per 1000**
**Overall**	42,861	176.0	1,705,316	7.0	42,355	173.3	1,670,148	6.8
**15-19**	446	21.1	26,828	1.3	465	22.0	28,151	1.3
**20-29**	9,320	204.9	497,623	10.9	8,714	191.8	464,435	10.2
**30-39**	12,226	283.0	552,009	12.8	12,271	280.8	553,526	12.7
**40-49**	9,052	222.9	326,776	8.0	8,987	222.1	324,318	8.0
**50-59**	8,507	196.0	235,227	5.4	8,110	189.4	223,680	5.2
**60-74**	3,310	66.6	66,853	1.3	3808	74.9	76,036	1.5
Males	29,692	246.3	1,160,021	9.6	29,520	244.0	1,138,053	9.4
**15-19**	313	29.0	18,361	1.7	310	28.8	18,293	1.7
**20-29**	6,789	292.0	353,600	15.2	6,280	270.5	326,405	14.1
**30-39**	8,788	404.9	386,488	17.8	8,771	399.0	385,142	17.5
**40-49**	6,142	305.0	214,399	10.6	6,250	311.5	218,542	10.9
**50-59**	5,496	259.0	145,464	6.9	5,281	252.1	139,492	6.7
**60-74**	2,164	92.3	41,709	1.8	2628	109.6	50,179	2.1
Females	13,169	107.1	545,295	4.4	12,835	104.0	532,095	4.3
**15-19**	133	12.9	8,466	0.8	155	15.0	9,859	1.0
**20-29**	2,531	113.8	144,023	6.5	2434	109.6	138,031	6.2
**30-39**	3,438	159.9	165,521	7.7	3,500	161.2	168,384	7.8
**40-49**	2,910	142.1	112,377	5.5	2,737	134.2	105,776	5.2
**50-59**	3,011	135.7	89,764	4.0	2,829	129.3	84,188	3.8
**60-74**	1146	43.7	25,144	1.0	1180	44.0	25,857	1.0

Note: Due to suppressed cells for some age-groups in the CDC-Wonder Database (when the cell represents fewer than 10 persons), total estimates of opioid toxicity deaths reported here may be a slight underestimate of the annual counts accessible in the CDC Wonder Database (since suppressed cells were imputed with a zero value in these calculations).

© 2023 Gomes T et al. *JAMA Network Open.*
