# Supplementary material for: Trends in Opioid Toxicity–Related Deaths in the US Before and After the Start of the COVID-19 Pandemic, 2011-2021
Source: JAMA Netw Open. 2023 Jul 7;6(7):e2322303. doi: 10.1001/jamanetworkopen.2023.22303 (PMC10329206; doi:10.1001/jamanetworkopen.2023.22303)
Supplement: Supplement 2. — Data Sharing Statement [file jamanetwopen-e2322303-s002.pdf]

Data Sharing Statement

Gomes. Trends in Opioid Toxicity–Related Deaths in the US Before and After the Start of the COVID-19 Pandemic, 2011-2021. *JAMA Netw Open*. Published July 07, 2023. doi:10.1001/jamanetworkopen.2023.22303

Data
Data available: No

Additional Information
Explanation for why data not available: Data are all aggregated and available through publicly accessible sources.
